# To be or not to be a virus: A novel chimeric circular Rep-encoding single stranded DNA virus with interfamilial gene exchange illustrates the considerable evolutionary capacity of ssDNA viruses

**DOI:** 10.1371/journal.pone.0309278

**Published:** 2025-08-18

**Authors:** Sélim Ben Chéhida, Sylvain Lacroix, Murielle Hoareau, Babbitha Fenelon, Arvind Varsani, Darren P. Martin, Pierre-Yves Teycheney, Pierre Lefeuvre, Jean-Michel Lett

**Affiliations:** 1 CIRAD, UMR PVBMT, St Pierre, France; 2 The Biodesign Center for Fundamental and Applied Microbiomics, Center for Evolution and Medicine, School of Life Sciences, Arizona State University, Tempe, Arizona, United States of America; 3 Structural Biology Research Unit, Department of Integrative Biomedical Sciences, University of Cape Town, Rondebosch, Cape Town, South Africa; 4 Computational Biology Division, Department of Integrative Biomedical Sciences, Institute of Infectious Diseases and Molecular Medicine, University of Cape Town, Observatory, South Africa; Nuclear Science and Technology Research Institute, IRAN, ISLAMIC REPUBLIC OF

## Abstract

Viruses in the family *Geminiviridae* cause significant economic losses in numerous crops worldwide. Some geminiviruses are often associated with satellite DNA molecules, such as alphasatellites (familly *Alphasatellitidae*), that require the assistance of a helper virus for their transmission. Here, we report the discovery of a chimeric virus, tentatively named Cenchrus purpureus associated virus (CPAV), in *Cenchrus purpureus* plants in La Réunion. The genome of CPAV consists of a single component that is primarily geminivirus-like. It contains a *rep* gene phylogenetically most closely related alphasatellites. This *rep* gene is positioned upstream of, and in the same orientation as, the movement and capsid protein genes. Both of these genes are phylogenetically most related to members of the genus *Mastrevirus* (family *Geminiviridae*). We found that CPAV is associated in the field with Cenchrus purpureus mild streak virus (CPMSV). Using agroinfectious clones and insect transmission assays, we demonstrated that CPAV is able to initiate infections in *C. purpureus* but its ability to establish long-term infection and be insect transmitted is apparently facilitated by CPMSV. This raises the question of whether CPAV qualifies as an autonomous virus or rather a satellite-like element with partial autonomy. The chimeric nature of CPAV illustrates the interfamily gene exchange between circular ssDNA viruses and satellites and how such recombination events can blur the boundaries between viruses and subviral agents. These findings highlight the evolutionary plasticity of circular ssDNA viruses and suggest that chimerism may be a key mechanism driving the emergence of novel viral forms with modified pathogenicity and host range.

## Introduction

A large number of novel cressdnaviruses have been discovered and characterized using viral metagenomic approaches [1,2]. These viruses are classified in a phylum called *Cressdnaviricota* [3]. Their genomes encode a conserved replication initiator protein (Rep) involved in rolling circle replication (RCR) [4,5]. The most recently proposed evolutionary scenario for the origin of cressdnaviruses hypothesizes that they originated from recombination events between prokaryotic plasmids carrying *rep* genes and capsid genes from eukaryotic (+)RNA viruses [6].

Although cressdnaviruses have now been identified from various environments and hosts [1,2,7], the first of these viruses to be characterized were those belonging to the family *Geminiviridae* [8]: a family which presently comprises 14 genera (*Becurtovirus*, *Begomovirus*, *Capulavirus*, *Citlodavirus, Curtovirus*, *Eragrovirus*, *Grablovirus*, *Maldovirus, Mastrevirus*, *Mulcrilevirus, Opunvirus, Topilevirus, Topocuvirus*, and *Turncurtovirus*; [9]). Geminiviruses are prevalent in many tropical and subtropical crops and wild plants [10]. Their genomes are either monopartite and ~2,600−3,600 nucleotides (nt) in size, or bipartite, with two DNA molecules of ~2,600 nt each, termed DNA-A and DNA-B [11]. All genomic DNAs share a virion-strand origin of replication nonanucleotide motif ‘TAATATT^↓^AC’, regardless of whether they have a monopartite or bipartite organization [9]. They are transmitted by insect vectors, such as leafhoppers (mastreviruses, becurtoviruses, turncurtoviruses), aphids (capulaviruses), whiteflies (begomoviruses), or treehoppers (topocuviruses and grabloviruses).

Geminiviruses, and some other cressdnaviruses in the families *Nanoviridae* and *Metaxyviridae*, are associated with circular single stranded DNA molecules called satellite DNAs. Unlike autonomous viruses, which can complete their infection cycle independently, satellite DNAs lack essential functions and rely on helper viruses for key processes such as replication, movement, transmission, and/or encapsidation [12]. These satellite DNAs belong to four genetically distinct groups: alphasatellites (family *Alphasatellitidae*), betasatellites (family *Tolecusatellitidae*), deltasatellites, and gammasatellites (for review, see [13–15]). The family *Alphasatellitidae* is subdivided in three subfamilies, *Geminialphasatellitinae*, *Nanoalphasatellitinae,* and *Petromoalphasatellitinae,* to accommodate the geminivirus-, nanovirus-, and metaxyvirus-associated alphasatellites, respectively. Satellite DNAs can modulate the pathogenicity of helper viruses, by enhancing (*e.g.,* betasatellites) and/or reducing (*e.g.,* alphasatellites and deltasatellites) both the accumulation of the helper virus and symptoms [12].

Alphasatellites encode a Rep and have a predicted stem-loop structure with, in most cases, the nonanucleotide sequence TAGTATT^↓^AC forming part of the loop [16]. Alphasatellites rely on their helper virus for encapsidation, movement and vector transmission, but they replicate autonomously.

Satellite DNAs associated with geminiviruses in the genus *Mastrevirus* were first described ten years ago [17,18]. More recently, Claverie *et al.* (2020) [19] described the natural association between a geminialphasatellite, sorghum mastrevirus-associated alphasatellite (SMasA), and a maize streak virus (MSV). MSV is one of several mastrevirus species in the African streak virus group which contains viruses that are widespread in sub-Saharan Africa and surrounding islands (Comoros, Madagascar, Mayotte, Mauritius, La Réunion) that are transmitted by several species of leafhoppers of the genus *Cicadulina*, including *Cicadulina mbila* [20–27].

Here, we report a novel cressdnavirus, tentatively named Cenchrus purpureus associated virus (CPAV; tentative species name *Cenpurvirus borbonicus*), naturally associated with Cenchrus purpureus mild streak virus (CPMSV, *Mastrevirus purpurei* [28]) identified in elephant grass (*Cenchrus purpureus*, family Poaceae) in La Réunion. We show that CPAV has a chimeric genome that is derived from an alphasatellite and a mastrevirus. Using agro-inoculation of infectious clones and vector transmission, we demonstrated that CPAV is able to initiate infections in *C. purpureus* but may require the assistance of CPMSV to establish long-term infections. While CPAV’s ability to be transmitted by insects remains uncertain, its apparent dependence on CPMSV suggests that further experiments would be needed to resolve its biology.

## Materials and methods

### Sampling and DNA extraction

**Fig 1:**
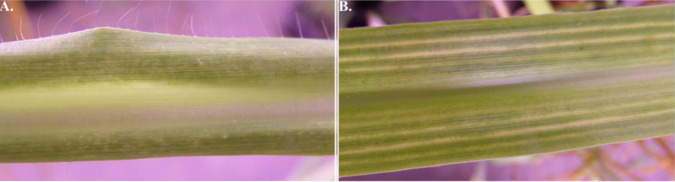
Leaves of *Cenchrus purpureus* plants from La Réunion showing (A) discontinuous chlorotic streaks and (B) continuous parallel chlorotic streaks, naturally infected by Cenchrus purpureus mild streak virus (CPMSV), and coinfected by CPMSV and Cenchrus purpureus associated virus (CPAV), respectively.

Three shoots were collected from three *Cenchrus purpureus* (elephant grass) plants in March 2021 at CIRAD’s (Centre de Coopération Internationale en Recherche Agronomique pour le Développement) Colimaçons experimental outstation (Latitude −21.1300; Longitude 55.3050) in Saint-Leu (La Réunion), where the study was conducted. The author was affiliated with CIRAD during the course of the study. As the research was conducted on CIRAD-owned land, no specific permits were required for field site access. The three shoots were labeled 21_REU_E0816_3, 21_REU_E0816_4, and 21_REU_E0816_5 and were found in the field showing discontinuous or continuous parallel chlorotic streak symptoms (**Fig 1**). All three collected shoots were potted in 2 L pots, each containing potting soil (Floradur BIO-SUBSTRAT, Floragard) and maintained in an insect-proof climatic chamber at 25°C, under 80% relative humidity and a 12-hr photoperiod. Symptomatic leaves were collected from each potted plant and grouped per plant, resulting in a total of three samples. Samples were dehydrated at 50°C for 48 hours and stored at room temperature until further use. Dehydrated leaf samples were homogenized using a TissueLyser II (Qiagen, Courtaboeuf, France) and total DNA was extracted from 20 mg of leaf material using the DNeasy Plant Pro kit (Qiagen, Courtaboeuf, France), following the manufacturer’s instructions. Purified DNA was stored at −20°C until further use.

### MinION sequencing and assembly

Sequence data were produced from the three total DNA extracts and two controls (DNA extracts from a tomato yellow leaf curl virus infected tomato plant and a healthy tomato plant being used as positive and negative control respectively) following the RCA-MinION protocol described by Ben Chéhida *et al.* [29] but using EquiPhi29 DNA polymerase (ThermoFisher Scientific, Les Ullis, France) for the rolling circle amplification (RCA) step. Sequencing was multiplexed (*i.e.,* pooled and sequenced simultaneously) on a single Flongle (FLO-FLG001) using the R9 chemistry as described in Ben Chéhida *et al.* [29] with nine other samples unrelated to this study.

Full cressdnavirus genomic components were assembled as described in Ben Chéhida *et al.* [29], following high accuracy basecalling and demultiplexing using Guppy v4.2.2 (https://nanoporetech.com/). Sequence coverage was estimated after raw reads were mapped back to the assembled sequences using minimap2 v2.17 [30]. All contigs were subjected to a BLAST search against the NCBI nucleotide database for preliminary species assignment.

### Virus indexing

Pairs of primers were designed to recover a genomic segment of CPMSV, CPAV, and MSV isolates. PCR amplification was achieved using either the primer pair NV3_F185: 5’-TGGAAAGTGGTAATTCGCCC-3’ and NV3_R1061: 5’-CGACTCCACTCTGAACTTCC-3’ for amplifying a 876 nt-long segment of CPMSV, the primer pair NV4_F241: 5’-AGCTTTGTCCCTCTACTGGT-3’ and NV4_R690: 5’-AGGTTGTCTTACCCTCTGCT-3’ for amplifying a 449 nt-long segment of CPAV, or the primer pair MSV_F1875: 5’-GGGACTGACCTGGAAGATGT-3’ and MSV_R2625: 5’-CTATAAAACAAGGAACGGCGG-3’ for amplifying a 750 nt-long segment of MSV. Amplification was carried out using the GoTaq® Master Mix Kit (Promega Corporation, Madison, Wisconsin- USA) and the following conditions: an initial denaturation at 95°C for 3 min, 30 cycles at 95°C for 30 sec, 60°C for 30 sec, 72°C for 3 min, and a final extension step at 72°C for 5 min.

### Full genome cloning and Sanger sequencing

Total genomic DNA extractions of one of the co-infected plants were subjected to RCA-RFLP for full genome cloning and Sanger sequencing as described in Inoue-Nagata *et al.* [31]. Briefly, an RCA amplification was performed from total DNA extract as described above, and products were digested with the XbaI restriction enzyme to produce monomeric units of viral genomes. The presence of a unique XbaI restriction site in both target viruses was identified using DNASTAR Lasergene (Madison, WI) software. Unit length genomic molecules of approximately 2.7–2.8 kb were gel purified, ligated into pJET 1.2 cloning vector (Thermo Fisher Scientific) and sequenced by standard Sanger sequencing using primer walking approach at Macrogen Europe. Full-length virus genomes were then assembled using Geneious Prime 2022.2 (http://www.geneious.com).

### Phylogenetic analysis

Firstly, an initial NCBI BLASTn and BLASTx searches were performed for each individual genome sequence obtained by Nanopore. Following this, a search for potential ORFs was made using DNAMAN v.10.0 [32] with known virus-encoded homologues to predicted expression products being searched for using BLASTp. After genome structural annotation, done on UGENE v.43.0 [33] and a gene cluster comparison generated with clinker v0.0.29 [34] on the nearest sequences of viruses for each ORF, trees were constructed for each ORF product against reference sequences of members from the most similar genus (S1 and S2 Tables). All sequence sets were aligned using MAFFT v7.475 [35]. After selecting the best fitting amino acid substitution model using ModelFinder [36], maximum likelihood (ML) phylogenetic trees were inferred using FastTree2 [37] with a SH-like approximate likelihood ratio test [38] of branch supports. Trees were edited using Figtree 1.4.4. [39] and the “ape” R package [40].

### Construction of viral agroinfectious clones

CPAV and CPMSV agroinfectious clones were constructed as described by Martin *et al.* [41]. Briefly, head-to-tail partial tandem copies (1.2 mer) of CPAV and CPMSV genomes obtained using Sanger sequencing (3,361 and 3,392 nt, respectively) were synthesized using Epoch Life Science Inc. (USA), linearized using EcoRI (absent from the complete genome sequences of CPAV and CPMSV and flanked to the 1.2mer sequences) and cloned into the EcoRI site of the binary vector pCambia 2300 [42]. Recombinant plasmids were transformed into *Escherichia coli* strain DH5α by electroporation and then transferred into *Agrobacterium tumefaciens* strain C58 by triparental mating using *E. coli* strain HMB101 harboring the helper plasmid pRK2013 [43]. The nucleotide sequence of each construct was confirmed by Sanger sequencing performed by Macrogen Europe (Amsterdam, the Netherlands) using primer walking.

### Agroinoculation and evaluation of infectivity

Firstly, agroinoculations were performed on three batches of seedlings: 20 three-day-old maize seedlings raised from seeds (*Zea mays*, cv. Golden Bantam) and 20 *Cenchrus purpureus* seedlings raised from cuttings (indexed as healthy using PCR prior to handling) were inoculated with a clone of CPAV; another 20 maize seedlings and 20 *C. purpureus* seedlings were inoculated with a clone of CPMSV; and a final batch of 20 maize seedlings and 20 *C. purpureus* seedlings were inoculated with a mix of CPAV and CPMSV clones, prepared independently and mixed immediately prior to agroinoculation. As described in Martin *et al.* [41], a standardized inoculum was prepared from actively growing *Agrobacterium tumefaciens* cultures adjusted to an optical density at 600nm of 0.4. The cultures were concentrated 10-fold after two washes with sterile distilled water and stored on ice for no more than 30 minutes before injection. Subsequently, 20 µl of inoculum were injected using a micro-fine syringe (BD Micro-Fine U-100 Insulin Syringe, 30G, BD Medical, Le Pont-de-Claix, France). Moreover, *A. tumefaciens* containing an agro-infectious clone of MSV-A [R2] [44] was used as a positive control of agroinoculation, whereas LB culture medium and *A. tumefaciens* devoid of recombinant binary vector were used as negative controls of agroinoculation. Finally, ten individuals of each plant species were used as non-inoculated negative controls (**Table 1**). A second batch of 89 seedlings of *C. purpureus* raised from cuttings of a plant grown from seeds was similarly processed with *A. tumefaciens* containing an infectious clone of CPAV. Five individuals of *C. purpureus* were used as non-inoculated negative controls (**Table 1**). All plants were potted in 0.75-liter pots, each containing potting soil (Floradur BIO-SUBSTRAT, Floragard) and maintained in an insect-proof growth climatic chamber as described above. Symptoms were monitored visually at 35 days post inoculation (dpi) and virus indexing was performed by PCR on leaves using specific primer pairs (S3 Table), as described above. Plants indexed as positive for CPMSV and/ or CPAV at 35 dpi were potted in 2 L pots and indexed again at 70 dpi and at 140 dpi.

**Table 1:** Infectivity of CPMSV, CPAV and MSV in single or mixed association after agroinoculation of *Cenchrus purpureus* and *Zea mays*. Percentage of infected plants was evaluated based on viral DNA detection by PCR at 35-, 70- and 140-days post inoculation. Proportions are indicated between brackets.

Host species/ inoculum	Trials	Days post inoculation (dpi)	Mock	*A. tumefaciens*	MSV-A [R2]	CPMSV	CPAV	CPMSV+CPAV
*Cenchrus purpureus*		35	0% (0/5)	0% (0/5)	0% (0/10)	0% (0/20)	11% (2/18)	0% (0/20)
	Trial 1	70	–	–	–	–	50% (1/2) *	–
		140	–	–	–	–	0% (0/1) ^#^	–
	Trial 2	30	0% (0/5)	0% (0/5)	–	–	1% (1/89)	–
		70	–	–	–	–	0% (0/1) *	–
*Zea mays*			0% (0/5)	0% (0/5)	50% (10/20)	0% (0/20)	0% (0/20)	0% (0/20)

*: Plants indexed positive by CPAV at 35dpi was tested at 70dpi.

^#^: Plant indexed positive by CPAV at 70dpi was tested at 140dpi.

-: Not available.

### Insect transmission assays

Transmission of CPAV and CPMSV by the leafhopper species, *Cicadulina mbila* (Hemiptera: Cicadellidae), which is one of the main vectors of African streak viruses in Africa [45], was evaluated. A non-viruliferous colony of *C. mbila* was reared on pearl millet plants (*Pennisetum glaucum*) in an insect-proof climatic growth chamber at 25°C, under 80% relative humidity and a 12-hr photoperiod. Viruliferous insects were obtained after a 72-h acquisition access period (AAP) performed in an insect-proof experimental cage 60x60x60 cm (Bugdorm 6E610, Wildcare Europe, Alès, France) either on field-collected *C. purpureus* plants co-infected by CPAV and CPMSV or on maize plants agroinfected by the MSV-A infectious clone used previously as a positive control for agroinoculation experiments (**Table 2**). Following the AAP, approximately 1,000 insects were transferred on *C. purpureus*, maize, sugarcane (*Sacharum officinarum*, cv. R570) and *Cenchrus echinatus* seedlings (indexed as healthy prior to handling by PCR diagnostic) potted in 0.75-liter pots each containing potting soil (Floradur BIO-SUBSTRAT, Floragard), for a 72-h inoculation access period (IAP). Then, insects were manually removed, and seedlings were sprayed with a systemic insecticide (Confidor®, Bayer). Mock-inoculated plants, using non-viruliferous leafhoppers, were used as negative controls. This experiment was conducted twice.

**Table 2:** Transmission rates of CPMSV, CPAV and MSV by *Cicadulina mbila* on four host species. Experiments were conducted twice with *C. mbila* after viral acquisition on co-infected *Cenchrus purpureus* plants collected in the field. Transmission rates were evaluated based on viral DNA detection by PCR at 35- and 70-days post inoculation (dpi). Proportions are indicated between brackets.

Host species	Days post inoculation (dpi)	Mock	MSV-A [R2]	Trial A			Trial B		
				**CPMSV**	**CPAV**	**CPMSV+CPAV**	**CPMSV**	**CPAV**	**CPMSV+CPAV**
*Cenchrus purpureus*	35	0% (0/5)	0% (0/13)	6% (1/17)	12% (2/17)	6% (1/17)	0% (0/15)	0% (0/15)	0% (0/15)
	70	–	–	0% (0/17)	0% (0/17)	12% (2/17)	–	–	–
*Zea mays*	35	0% (0/5)	100% (5/5)	0% (0/8)	0% (0/8)	0% (0/8)	0% (0/6)	0% (0/6)	0% (0/6)
*Cenchrus echinatus*	35	0% (0/5)	–	0% (0/5)	0% (0/5)	0% (0/5)	0% (0/5)	0% (0/5)	0% (0/5)
*Saccharum officinarum*	35	0% (0/5)	0% (0/13)	0% (0/10)	0% (0/10)	0% (0/10)	0% (0/10)	0% (0/10)	0% (0/10)

-: Not available.

Plants were maintained in the same growth conditions as those described above. After 30 days, symptoms of infection were recorded on, and the presence of viral DNA was assessed in new developed leaves using PCR and specific primer pairs (S3 Table). All plants were potted in 2 L pots and retested at 70 dpi.

## Results

### Detection of a novel virus infecting C. purpureus

A total of 176,330 raw reads were obtained from the three analyzed *C. purpureus* samples using the RCA-MinION workflow (S4 Table). Of these, 125,778 (71%) barcoded reads passed the quality control. The three barcodes associated with *C. purpureus* samples were retrieved from 10,616 (8%) reads, 19,498 (16%) reads, and 10,352 (8%) for each, respectively. The median read lengths for the three barcodes were 335 nt, 177 nt, and 441 nt, with longest reads of 7,706 nt, 8,272 nt, and 5,720 nt, respectively. Two complete viral genomes were assembled: (1) Cenchrus purpureus mild streak virus (CPMSV [28]); and (2) a novel cressdnavirus named Cenchrus purpureus associated virus (CPAV). Both viruses were present in samples 21_REU_E0816_3 and 21_REU_E0816_4 whereas only CPMSV was detectably present in sample 21_REU_E0816_5 (S4 Table).

The two CPAV genome sequences assembled from samples 21_REU_E0816_3 and 21_REU_E0816_4 with 1,543 and 677 MinION reads, were 2,741 and 2,725 nt long, respectively and shared 98.8% genome-wide pairwise nucleotide identity, whereas the three CPMSV genome sequences assembled from samples 21_REU_E0816_3, 21_REU_E0816_4 and 21_REU_E0816_5 from 325, 455 and 90 MinION reads, were 2,811, 2,804 and 2,797 nt long, respectively (S4 Table) and shared between 98.7% and 99% genome-wide pairwise nucleotide identity.

A full CPAV genome sequence of 2,734 nt was amplified by RCA, cloned, and Sanger sequenced from sample 21_REU_E0816_3 (GenBank accession number OQ451138). This 2,734 nt Sanger sequence shared 99.4% and 99.6% genome-wide pairwise nucleotide identity with the MinION sequences assembled from samples 21_REU_E0816_3 and 21_REU_E0816_4, respectively. This Sanger sequence was considered the sequence of the exemplar isolate of CPAV and used in further molecular characterization of the CPAV genome. An agroinfectious clone of the CPAV genome was constructed from the 2,734 nt Sanger sequence and was found to be infectious upon agroinoculation of *C. purpureus* plants. Similarly, a CPSMV genome sequence of 2,808 nt was amplified, cloned, and Sanger sequenced from sample 21_REU_E0816_5 (GenBank accession number OQ455385). This Sanger sequence shared 99.1%, 99.5%, and 99.2% genome-wide pairwise nucleotide identity with the MinION sequences assembled from samples 21_REU_E0816_3, 21_REU_E0816_4, and 21_REU_E0816_5, respectively.

### CPAV is a novel virus that is mastrevirus-like with a chimeric genome organization

**Fig 2:**
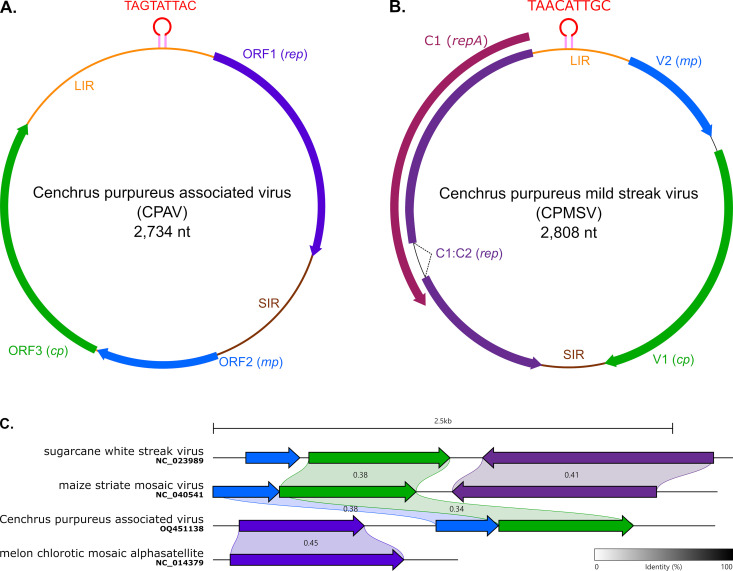
Schematic genome organizations of (A) Cenchrus purpureus associated virus (CPAV) and (B) Cenchrus mild streak virus (CPMSV). Open reading frames are shown with colored arrows and intergenic regions with thin colored lines. The stem-loop and nonanucleotide motif sequence are also shown. (C) Gene cluster comparison of CPAV with the nearest sequences of viruses for each ORFs.

**Fig 3:**
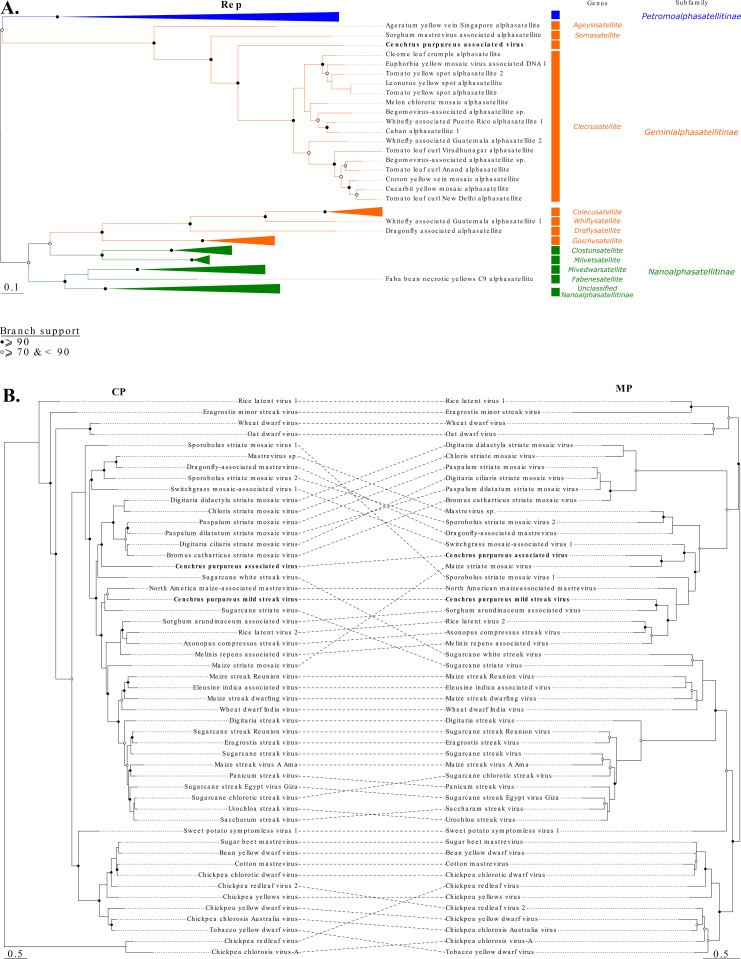
(A) Unrooted maximum likelihood tree inferred from aligned replication associated protein (Rep) amino acid sequences of viruses in family *Alphasatellitidae*. (B) On the left, unrooted maximum likelihood tree inferred from aligned capsid protein (CP) and on the right, unrooted neighbor-joining tree inferred from the aligned movement protein (MP) amino acid sequences of representative mastreviruses. CPAV is shown in bold font. Open and closed circles on nodes indicate bootstrap support for the branches to their left of 70-89% and ≥90% respectively.

The complete genome sequence of 2,734 nt of CPAV was subjected to a BLASTn analysis for preliminary identification. It revealed hits with alphasatellites including a 422 nt region with the highest nucleotide identity (68.48%) with Croton yellow vein mosaic alphasatellite (MZ622156). Analysis of the genomic organization revealed three non-overlapping open reading frames (ORFs) all in the same orientation (Figs 2A and 2C). ORF1 encodes a putative replication-associated protein (Rep, 309 aa) most closely related to the Rep of melon chlorotic mosaic alphasatellite (YP_003828902), with 64% aa similarity. The putative CPAV Rep shares the conserved rolling circle replication (RCR) motifs (motif I: CFTLFE; motif II: KHLQG; motif III: YCTK) and superfamily 3 helicase (SF3) motifs (Walker-A: GSVGAEGKT; Walker-B: CVFDL; Motif C: VFAN) of alphasatellite replication-associated proteins (Rosario *et al.*, 2012; S1 Fig). ORF2 encodes a putative movement protein (MP, 113 aa) most closely related (49% aa similarity) to the MP of the mastrevirus, maize striate mosaic virus (YP_009551895). ORF3 encodes a putative coat protein (CP, 244 aa) most closely related (35% aa similarity) to the CP of the mastrevirus, sugarcane white streak virus (YP_009026387). The end of ORF 1 and the start of ORF2 is separated by a short intergenic region (SIR, 144 nt) and the end of ORF 3 and the start of ORF1 is separated by a long intergenic region (LIR, 589 nt). We failed to detect any nucleotide sequence similarities to these intergenic regions in any other viruses using BLASTn (**Fig 3**). However, the CPAV LIR includes a stem-loop structure in the LIR at a position analogous to the origin of virion strand replication in geminiviruses. The loop of this structure has the canonical nonanucleotide motif (5’-TAGTATT^↓^AC-3’) of geminialphasatellites [16]. The 5’ end of the CPAV LIR displays a distinctive adenine rich region (A-rich region) that is also characteristic of geminialphasatellites [16] at its 5’ end (S1 Fig). Lastly, TATA and GC boxes of the presumed Rep and MP-CP promoters were identified upstream of ORFs 1 and 2, respectively.

While phylogenetic analysis using Rep amino acid sequences placed CPAV within the subfamily *Geminialphasatellitinae* of the family *Alphasatellitidae*, it was too divergent from currently known geminiaphasatellites to be credibly assigned to any particular established genus (**Fig 3A**). A similar analysis performed on either the CPAV MP or the CPAV CP amino acid sequences placed CPAV in the genus *Mastrevirus* (**Fig 3B**), suggesting that the genome of CPAV has a chimeric structure incorporating ORF1 from a geminialphasatellite and ORFs 2 and 3 from a mastrevirus.

The complete genome of CPMSV (**Fig 2B**) shares its highest degree of nucleotide identity (57.9%) with that of Sorghum arundinaceum associated virus (MK546381), a mastrevirus. Its organization was typical of mastreviruses with two ORFs on the virion-sense strand likely encoding a movement protein (55% similar to that of Sorghum arundinaceum associated virus YP_010087741) and a capsid protein (52% similarity to that of sugarcane striate virus YP_009389275), respectively, and two overlapping ORFs on the complementary-sense strand likely encoding a replication-associated protein and a RepA protein (50% similar to those of Sporobolus striate mosaic virus 2 YP_006666531). However, the genome of CPMSV contains an unusual nonanucleotide sequence, TAACATT^↓^GC, within the loop of a stem-loop structure located in the large intergenic region that contains the presumed virion strand origin of replication.

### Infectivity of CPAV and CPMSV infectious clones

Given that CPAV is an unprecedented viral entity, we attempted to determine whether it is infectious either on its own or in conjunction with CPMSV as a helper-virus. In a first infection trial, 11% (2/18) of *C. purpureus* plants agroinoculated with CPAV tested positive by PCR at 35 dpi and 6% (1/18) at 70 dpi but were negative at 140 dpi, and remained asymptomatic throughout the experiment (**Table 1**). In a second infection trial, 1% (1/89) of *C. purpureus* plants agroinoculated with CPAV tested positive by PCR at 30 dpi, which was negative at 70 dpi, and remained also asymptomatic (**Table 1**). Viral infection was assessed by PCR using specific primers (S3 Table), and by direct Sanger sequencing of PCR amplicons, which confirmed the specificity of the viral diagnosis. None of the *C. purpureus* plants agroinoculated with CPMSV or CPMSV+CPAV tested positive by PCR. For the control condition, 50% (10/20) of maize plants agroinoculated with the MSV-A infectious clone tested positive for MSV-A and presented with leaf streak symptoms at 35 days post inoculation (dpi).

### Transmission capacity of CPAV and CPMSV by leafhoppers

To determine the transmissibility of CPAV and/or CPMSV by *C. mbila*, we used coinfected *C. purpureus* plants 21_REU_E0816_3 and 21_REU_E0816_4, collected in the field. At 35 dpi, 12.5% (4/32; trials A and B) of the *C. purpureus* plants were indexed positive by PCR either for CPMSV (1 plant), CPAV (2 plants) or jointly CPAV and CPMSV (1 plant) (**Table 2**). All four infected plants were asymptomatic. PCR amplicons obtained for CPAV showed a more intense band when the virus was in coinfection with CPMSV (S2 Fig). At 70 dpi, the two plants previously indexed positive for CPAV became negative while the plant co-infected by CPAV and CPMSV remained positive for both viruses. However, the plant found to be infected by CPMSV alone at 35 dpi was indexed positive by PCR for both CPAV and CPMSV at 70 dpi. All positive PCR results were confirmed by sequencing the amplicons. For the virus-vector control combination, all five *Z. mays* plants used for transmission of MSV-A by *C. mbila* were indexed positive by PCR and showed severe leaf streak symptoms. Conversely, none of the *C. echinatus* and sugarcane plants used in a similar insect transmission assay developed noticeable symptoms and none were found to be infected by MSV-A, CPMSV, or CPAV.

## Discussion

### Discovery of an unusual mastrevirus-alphasatellite chimera

Here we describe a novel virus that is mastrevirus-like, CPAV, that has a genome architecture that is unique among the known geminiviruses. Whereas the CPAV replication associated protein gene and LIR are most closely related to those of alphasatellites (family *Alphasatellitidae*), its movement and capsid protein genes are most closely related to those of mastreviruses (family *Geminiviridae*). Interestingly, the CPAV *rep* gene and LIR together are 1,516 nt long: a size that is well within the size range of alphasatellite genomes, suggesting that this peculiar viral genome could have descended from an alphasatellite that acquired and domesticated the *mp* and *cp* genes from a currently undescribed mastrevirus. Inter-family recombination between alphasatellites and begomoviruses has been reported previously [46,47]. These findings suggest that both ssDNA viruses and satellites, despite their evolutionary divergence, have the ability to exchange their genetic information, and that the resulting recombinant architecture carries a functional replication protein, enabling these recombinant viruses to independently replicate within host cells and, probably, also move from cell-to-cell.

### Evolution of circular ssDNA viruses

CPAV adds to a growing list of chimeric cressdnaviruses [48–52], providing further evidence that inter-familial horizontal gene transfer (HGT) has been a major contributor to the genesis of new genera- and family-level groupings within the *Cressdnaviricota* phylum [51]. HGT is known to play a key role in the evolution of viruses and can occur through either homologous or non-homologous recombination processes [53,54]. Although the propensity of cressdnaviruses to recombine through recombination-dependent replication (RDR; [55,56]) is most probably paramount for the emergence of viruses with mixed origin, most of these horizontal exchanges would certainly result in detrimental and negatively selected combinations, later purged from viral populations [57,58]. In the case of CPAV, it is likely that the relative conservation of an alphasatellite backbone would facilitate the integration of other ORFs into a functional ensemble.

### Is CPAV a genuine autonomous virus?

The *ca.* 2.8kb genome size and the presence of a CP similar in sequence and size to those of mastreviruses would suggest that CPAV forms twinned quasi-icosahedral particles [59] and is transmitted similarly to mastreviruses. Despite the small sample size, transmission assays demonstrated that CPAV was transmitted by the leafhopper *C. mbila.* However, we were only able to detect transmission from field sampled plants that were coinfected with CPAV and CPMSV. Giving that viruses on vegetatively propagated plants generally have lower insect transmission efficiency compared to those on seasonal plants [60], establishing controlled infections of these viruses from only a single cloned genome for each virus has proved difficult, and controlled co-infections even more so. We were therefore unable to rigorously test whether CPAV is transmissible by leafhopper in the absence of CPMSV.

In the small number of instances where we were able to initiate controlled infections of *C. purpureus* from cloned CPAV and CPMSV genomes, it seems that CPAV may require CPMSV and/or an additional component to sustain infections lasting longer than ~70 days. However, it cannot be excluded that CPAV genome titers may have simply decreased by 70 dpi to the point where they dropped below the PCR detection threshold, a situation that is quite commonly encountered with some geminiviruses [61], and could be attributed to the triggering of post transcriptional gene silencing (PTGS)-based host defense mechanisms [62]. Additional analyses, such as investigating the presence of viral short interfering RNAs [63,64], are needed to determine whether CPAV infections trigger PTGS in *C. purpureus*.

It is noteworthy that CPMSV infections could not be experimentally established in *C. purpureus* following agroinoculation of a CPMSV infectious clone, possibly due to the suboptimal quality of the inoculum source, but proved successful following insect transmission from a plant co-infected with CPMSV and CPAV. This result suggests that CPAV could potentially act as the helper virus when plants are coinfected with CPMSV: at least in terms of infection initiation or transmission, although further data are needed to confirm this hypothesis. Mutualistic interactions, which are known to promote viral synergism and the evasion of host resistance [65–68], were previously reported for members of the *Geminiviridae* family in different situations. They include both coinfections by a luteovirus (barley yellow dwarf virus) and a mastrevirus (wheat dwarf virus) in wheat [68], coinfections by two viral components of a begomovirus such as pepper yellow vein Mali virus DNA-A and DNA-B in tomato [69], or coinfections of a begomovirus (Euphorbia yellow mosaic virus) and an alphasatellite (Euphorbia yellow mosaic alphasatellite) in *Euphorbia heterophylla* plants [70].

Importantly, no part of the CPAV genome displayed a close similarity to any known virus sequence available in the GenBank database, including ssDNA viruses with circular genomes that were identified recently in La Réunion through circomics approaches [71,72], suggesting that the parental viruses of CPAV are not highly prevalent in La Réunion or may even be extinct. The detection of CPAV from multiple plant samples provides a strong indication that CPAV is not a transient artifact viral construct, but rather represents an actual circulating virus that is successfully occupying an ecological niche. While our RCA-MinION approach specifically targets circular DNA viruses, an unbiased NGS approach would be necessary to rule out the presence of other viruses, such as RNA viruses, potentially associated with the observed symptoms. Additionally, a follow-up using quantitative PCR could provide valuable insights into the potential synergistic effects of CPAV and CPMSV, further elucidating their interactions and impact on *C. purpureus*. Further studies and large-scale surveys of C. purpureus in La Réunion and elsewhere are now needed to investigate not only the prevalence, host range, and ecological context of this novel entity, but also to address the central question it raises about whether CPAV should “be or not be” considered as a virus. The possibility of sequencing historical viral genomes from herbarium specimens also represents an opportunity to investigate the parental lineages of these “viruses” and their evolutionary history [73].

## Supporting information

S1 Fig Nucleotide sequence of Cenchrus purpureus associated virus linearized at the nick site (^↓^) of conserved nonanucleotide sequence TAGTATT^↓^AC (highlighted in red) located at the stem loop (highlighted in pink). Replication associated protein gene (*rep*), movement protein gene (V2, *mp*) and capsid protein gene (V1, *cp*) are respectively highlighted in purple, blue and green. The amino acid sequence of the three ORFs are shown and the start codon is in bold. Conservative RCR and SF3 helicase amino acid motives are respectively highlighted in orange and brown and the conserved amino acid residues are in bold. ‘RRR’ indicates the presence of an N-terminal region rich in basic amino acids. TATA and GC boxes are respectively highlighted in light blue and gray. Long intergenic region (LIR) and short intergenic region (SIR) sequences are respectively in dark red and dark brown font. The A-rich region of the LIR is highlighted in yellow.
(TIFF)

S2 Fig Electropherograms of PCR products from total DNA extracts of *Cenchrus purpureus* plants (samples A15 and A16) at 30 days post-inoculation following transmission assays of Cenchrus purpureus associated virus (CPAV) and Cenchrus purpureus mild streak virus (CPMSV) using *Cicadulina mbila* from coinfected plants. Positive controls (T+ CPAV and T+ CPMSV) are included. High-resolution capillary electrophoresis was performed using the QIAxcel system (QIAGEN, Germany). The size markers (in base pairs) are displayed on the left and right axes, with prominent peaks corresponding to the expected amplicon sizes for each virus.
(TIFF)

S1 Table List of the Alphasatellitidae species with the accession numbers of the nucleotide complete and amino acid replication associated protein (Rep) sequences. (DOCX)

S2 Table List of the Mastrevirus species with the accession numbers of the nucleotide complete, amino acid movement protein (MP) and capsid associated protein (CP) sequences. (DOCX)

S3 Table Primers used for virus indexing. (DOCX)

S4 Table Long read sequencing statistics. (DOCX)
