# Supplementary figures and images for: To be or not to be a virus: A novel chimeric circular Rep-encoding single stranded DNA virus with interfamilial gene exchange illustrates the considerable evolutionary capacity of ssDNA viruses
Source: PLoS One. 2025 Aug 18;20(8):e0309278. doi: 10.1371/journal.pone.0309278 (PMC12360566; doi:10.1371/journal.pone.0309278)

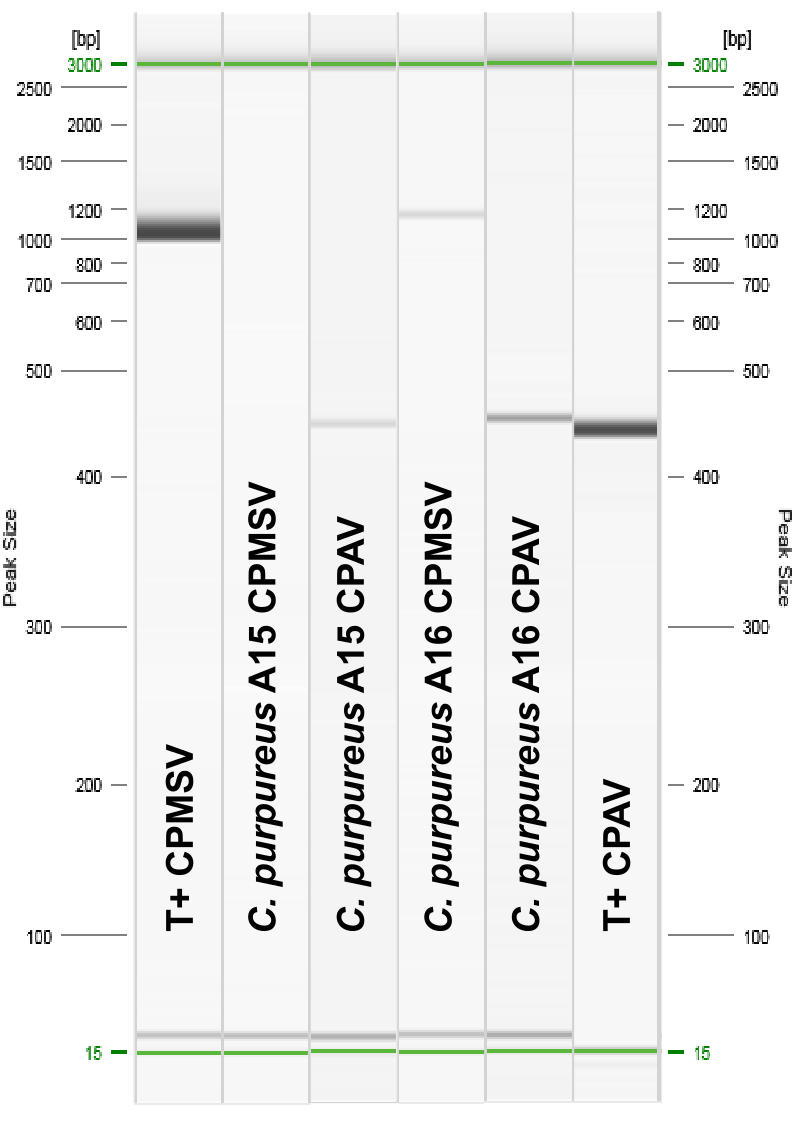

Supplement: S2 Fig — Positive controls (T+ CPAV and T+ CPMSV) are included. High-resolution capillary electrophoresis was performed using the QIAxcel system (QIAGEN, Germany). The size markers (in base pairs) are displayed on the left and right axes, with prominent peaks corresponding to the expected amplicon sizes for each virus. (TIFF) [file pone.0309278.s002.tiff]
