# Supplementary material for: To be or not to be a virus: A novel chimeric circular Rep-encoding single stranded DNA virus with interfamilial gene exchange illustrates the considerable evolutionary capacity of ssDNA viruses
Source: PLoS One. 2025 Aug 18;20(8):e0309278. doi: 10.1371/journal.pone.0309278 (PMC12360566; doi:10.1371/journal.pone.0309278)
Supplement: S1 Table — (DOCX) [file pone.0309278.s003.docx]

**Supplementary Table 1. List of the *Alphasatellitidae* species with the accession numbers of the nucleotide complete and amino acid replication associated protein (Rep) sequences.**

| ***Alphasatellitidae* species** | **Complete** | **Rep** |
| --- | --- | --- |
| Tomato leaf curl Pakistan alphasatellite | FM164939 | YP_002941920 |
| Parthenium leaf curl alphasatellite | LN906596 | YP_009254744 |
| Begomovirus-associated alphasatellite sp. TW | KU923760 | AOY34818 |
| Leonurus yellow spot alphasatellite | KX348231 | AQT01637 |
| Tomato leaf curl New Delhi alphasatellite | MH550542 | QAB08755 |
| Begomovirus-associated alphasatellite sp. UHAsV-1.CD-W | MN203219 | QHB15188 |
| Sorghum mastrevirus associated alphasatellite | MN901968 | QKK13640 |
| Tomato yellow spot alphasatellite 2 | MN518743 | YP_010087853 |
| Parsley severe stunt alphasatellite 4 | MK039142 | YP_010087622 |
| Parsley severe stunt alphasatellite 3 | MK039141 | YP_010087621 |
| Tomato leaf curl Anand alphasatellite | MH577036 | YP_010087615 |
| Faba bean necrotic yellows virus associated alphasatellite 1 | MF510471 | YP_010086795 |
| Tomato leaf curl Virudhunagar alphasatellite | KY848691 | YP_010086787 |
| Eclipta yellow vein alphasatellite | KX938425 | YP_010086775 |
| Coconut foliar decay alphasatellite 5 | MF926430 | YP_010087372 |
| Coconut foliar decay alphasatellite 4 | MF926429 | YP_010087370 |
| Coconut foliar decay alphasatellite 2 | MF926426 | YP_010087364 |
| Coconut foliar decay alphasatellite 7 | MF926432 | YP_009553579 |
| Coconut foliar decay alphasatellite 6 | MF926431 | YP_009553054 |
| Coconut foliar decay alphasatellite 3 | MF926427 | YP_009552699 |
| Tomato yellow leaf curl China alphasatellite | AM749493 | YP_009666132 |
| Banana bunchy top alphasatellite 2 | AF416471 | YP_009508281 |
| Whitefly associated Guatemala alphasatellite 1 | KT099172 | YP_009508355 |
| Banana bunchy top alphasatellite 3 | HQ616080 | YP_009508283 |
| Banana bunchy top alphasatellite 1 | L32167 | YP_009508165 |
| Sida leaf curl alphasatellite | FR772088 | YP_009513247 |
| Malvastrum yellow mosaic alphasatellite | AM236765 | YP_009513246 |
| Chilli leaf curl alphasatellite | KF471043 | YP_009513231 |
| Vernonia yellow vein Fujian alphasatellite | KC959931 | YP_009513230 |
| Gossypium darwinii symptomless alphasatellite | EU384623 | YP_009513229 |
| Tomato yellow leaf curl Yunnan alphasatellite | KX759649 | YP_009513233 |
| Hollyhock yellow vein virus associated symptomless alphasatellite | FR772086 | YP_009508886 |
| Bendhi Yellow Vein Mosaic/Mesta Yellow Vein Mosaic alphasatellite | FN658716 | YP_009508885 |
| Nanovirus-like particle Egypt | AJ512960 | YP_009508879 |
| Nanovirus-like particle Singapore | AJ238493 | YP_009508878 |
| Nanovirus-like particle Pakistan | AJ132344 | YP_009508877 |
| Sophora alopecuroides yellow stunt alphasatellite 9 | KX534409 | YP_009508448 |
| Sophora alopecuroides yellow stunt alphasatellite 4 | KX534408 | YP_009508447 |
| Sophora alopecuroides yellow stunt alphasatellite 7a | KX534400 | YP_009508446 |
| Sophora alopecuroides yellow stunt alphasatellite 1a | KX534399 | YP_009508445 |
| Sophora alopecuroides yellow stunt alphasatellite 8 | KX534398 | YP_009508444 |
| Tomato yellow spot alphasatellite | KX348228 | YP_009508434 |
| Whitefly associated Puerto Rico alphasatellite 1 | KT099173 | YP_009508356 |
| Whitefly associated Guatemala alphasatellite 2 | KT099170 | YP_009508354 |
| Pea necrotic yellow dwarf alphasatellite 3 | KC979052 | YP_009508292 |
| Pea necrotic yellow dwarf alphasatellite 1 | KC979051 | YP_009508291 |
| Gossypium mustelinum symptomless alphasatellite | EU384656 | YP_009508282 |
| Tomato leaf curl Cameroon alphasatellite CM:TOS2D1:To:09 | FN675299 | YP_009508213 |
| Tomato leaf curl Cameroon alphasatellite CM:OMHD3:Ok:09 | FN675296 | YP_009508212 |
| Okra yellow crinkle Cameroon alphasatellite | FN675284 | YP_004123952 |
| Ageratum yellow vein alphasatellite | KY001650 | YP_009344825 |
| Pedilanthus leaf curl alphasatellite | KX168428 | YP_009338004 |
| Tomato leaf curl alphasatellite | LN874304 | YP_009337831 |
| Hibiscus leaf curl alphasatellite | HG934827 | YP_009337830 |
| Ageratum yellow vein Pakistan alphasatellite | HG934822 | YP_009337826 |
| Cucurbit yellow mosaic alphasatellite | KT948075 | YP_009246456 |
| Bhindi yellow vein alphasatellite | KT716081 | YP_009230209 |
| Ageratum conyzoides symptomless alphasatellite | KT390508 | YP_009174978 |
| Sida yellow vein mosaic alphasatellite | KT390506 | YP_009174977 |
| Chilli leaf curl India alphasatellite | KF471037 | YP_009154718 |
| Synedrella leaf curl alphasatellite | KJ939346 | YP_009073582 |
| Faba bean necrotic yellows C1 alphasatellite | X80879 | YP_009058890 |
| Ageratum conyzoides associated symptomless alphasatellite | HG518788 | YP_009052483 |
| Sida yellow vein alphasatellite | KJ614231 | YP_009044083 |
| Faba bean necrotic stunt alphasatellite 1 | KC978991 | YP_009021881 |
| Faba bean necrotic stunt alphasatellite 2 | KC978990 | YP_009021880 |
| Black medic leafroll alphasatellite 1 | KC978957 | YP_009021872 |
| Sida yellow vein China alphasatellite | KC677735 | YP_009002582 |
| Ageratum yellow vein China alphasatellite | KF785752 | YP_009001905 |
| Cardamom bushy dwarf alphasatellite | KF435148 | YP_008854133 |
| Cuban alphasatellite 1 | HE806451 | YP_008169853 |
| Guar leaf curl alphasatellite | KC305096 | YP_007518505 |
| Ageratum enation alphasatellite | JX913532 | YP_007011043 |
| Ageratum yellow vein India alphasatellite | JX570736 | YP_007011042 |
| Sunflower leaf curl Karnataka alphasatellite | JX569789 | YP_007004041 |
| Dragonfly associated alphasatellite | JX458742 | YP_007004040 |
| Cassava mosaic Madagascar alphasatellite | HE984148 | YP_006742179 |
| Bhendi yellow vein mosaic virus-associated alphasatellite | JX183091 | YP_006666513 |
| Mesta yellow vein mosaic alphasatellite | JX183090 | YP_006666512 |
| Cotton leaf curl Multan alphasatellite | FR877532 | YP_006488615 |
| Cotton leaf curl Lucknow alphasatellite | HQ343234 | YP_004347413 |
| Malvastrum yellow mosaic Cameroon alphasatellite | FN675297 | YP_004123953 |
| Tomato leaf curl Cameroon alphasatellite | FR717142 | YP_004046695 |
| Cleome leaf crumple alphasatellite | FN436007 | YP_003987456 |
| Euphorbia yellow mosaic virus associated DNA 1 | FN436008 | YP_003966132 |
| Tobacco leaf curl PUSA alphasatellite | HQ180392 | YP_003934916 |
| Melon chlorotic mosaic alphasatellite | HM163578 | YP_003828902 |
| Croton yellow vein mosaic alphasatellite | FN658711 | YP_003433564 |
| Cotton leaf curl Burewala alphasatellite | FN658727 | YP_003433566 |
| Cotton leaf curl Gezira alphasatellite | FN554580 | YP_003334471 |
| Chilli leaf curl Multan alphasatellite | FM179614 | YP_003104752 |
| Gossypium davidsonii symptomless alphasatellite | EU384655 | YP_003082245 |
| Okra leaf curl alphasatellite Mali | EU589450 | YP_001856218 |
| Mimosa yellow leaf curl virus-associated DNA 1 | DQ641719 | YP_001285943 |
| Sida yellow vein Vietnam alphasatellite | DQ641718 | YP_001285945 |
| Okra leaf curl alphasatellite Pakistan | AJ512954 | YP_031730 |
| Tobacco curly shoot alphasatellite | AJ579345 | NP_878243 |
| Subterranean clover stunt C6 alphasatellite | U16735 | NP_620700 |
| Subterranean clover stunt C2 alphasatellite | U16731 | NP_620696 |
| Milk vetch dwarf C10 alphasatellite | AB009047 | NP_619768 |
| Milk vetch dwarf C3 alphasatellite | AB000922 | NP_619761 |
| Milk vetch dwarf C2 alphasatellite | AB000921 | NP_619760 |
| Milk vetch dwarf C1 alphasatellite | AB000920 | NP_619759 |
| Faba bean necrotic yellows C9 alphasatellite | AJ132187 | NP_619574 |
| Faba bean necrotic yellows C7 alphasatellite | AJ132185 | NP_619572 |
| Faba bean necrotic yellows C11 alphasatellite | AJ005968 | NP_619565 |
| Ageratum yellow vein Singapore alphasatellite | AJ416153 | NP_579867 |
| Coconut foliar decay alphasatellite | M29963 | NP_040944 |
