# Supplementary material for: To be or not to be a virus: A novel chimeric circular Rep-encoding single stranded DNA virus with interfamilial gene exchange illustrates the considerable evolutionary capacity of ssDNA viruses
Source: PLoS One. 2025 Aug 18;20(8):e0309278. doi: 10.1371/journal.pone.0309278 (PMC12360566; doi:10.1371/journal.pone.0309278)
Supplement: S2 Table — (DOCX) [file pone.0309278.s004.docx]

**Supplementary Table 2. List of the *Mastrevirus* species with the accession numbers of the nucleotide complete, amino acid movement protein (MP) and capsid associated protein (CP) sequences.**

| ***Mastrevirus* Species** | **Complete** | **CP** | **MP** |
| --- | --- | --- | --- |
| Axonopus compressus streak virus | KJ437671 | YP_009021762 | YP_009021761 |
| Bean yellow dwarf virus | Y11023 | NP_612220 | NP_612219 |
| Bromus catharticus striate mosaic virus | HQ113104 | YP_004089626 | YP_004089625 |
| Chickpea chlorosis Australia virus | JN989422 | YP_008472703 | YP_008472702 |
| Chickpea chlorosis virus | GU256530 | YP_004046666 | YP_004046665 |
| Chickpea chlorotic dwarf virus | AM850136 | YP_002014711 | YP_002014710 |
| Chickpea redleaf virus | GU256532 | YP_004046662 | YP_004046661 |
| Chickpea redleaf virus 2 | MK940528 | YP_010087771 | YP_010087770 |
| Chickpea yellow dwarf virus | KM377674 | YP_009104362 | YP_009104361 |
| Chickpea yellows virus | JN989439 | YP_009506576 | YP_009506575 |
| Chloris striate mosaic virus | M20021 | NP_040952 | NP_040951 |
| Cotton mastrevirus | HE956706 | CCI88379 | CCI88378 |
| Digitaria ciliaris striate mosaic virus | JQ948088 | YP_006666534 | YP_006666533 |
| Digitaria didactyla striate mosaic virus | HM122238 | YP_003915158 | YP_003915157 |
| Digitaria streak virus | M23022 | NP_040963 | NP_040962 |
| Dragonfly-associated mastrevirus | JX458741 | YP_007004037 | YP_007004036 |
| Eleusine indica associated virus | MK546379 | YP_010087739 | YP_010087738 |
| Eragrostis minor streak virus | JF508490 | YP_004465363 | YP_004465362 |
| Eragrostis streak virus | EU244915 | YP_001686793 | YP_001686792 |
| Maize streak dwarfing virus | MK329300 | YP_010087227 | YP_010087226 |
| Maize streak Reunion virus | JQ624879 | YP_006331072 | YP_006331071 |
| Maize streak virus | AF329878 | YP_009154762 | YP_009154761 |
| Maize striate mosaic virus | MF167299 | YP_009551896 | YP_009551895 |
| Mastrevirus sp. | MN203180 | QHB15156 | QHB15157 |
| Melinis repens associated virus | MK546380 | QDO73337 | QDO73336 |
| North American maize-associated mastrevirus | MZ852895 | ULE36139 | ULE36138 |
| Oat dwarf virus | AM296025 | YP_001941161 | YP_001941160 |
| Panicum streak virus | L39638 | NP_042589 | NP_042588 |
| Paspalum dilatatum striate mosaic virus | JQ948061 | YP_006666522 | YP_006666521 |
| Paspalum striate mosaic virus | JF905486 | YP_006659973 | YP_006659972 |
| Rice latent virus 1 | KY962374 | YP_009553474 | YP_009553473 |
| Rice latent virus 2 | KY962381 | YP_009551799 | YP_009551798 |
| Saccharum streak virus | GQ273988 | YP_003288767 | YP_003288766 |
| Sorghum arundinaceum associated virus | MK546381 | YP_010087742 | YP_010087741 |
| Sporobolus striate mosaic virus 1 | JQ948051 | YP_006666526 | YP_006666525 |
| Sporobolus striate mosaic virus 2 | JQ948052 | YP_006666530 | YP_006666529 |
| Sugar beet mastrevirus | EU034169 | ABV82711 | ABV82710 |
| Sugarcane chlorotic streak virus | KX787914 | YP_009325924 | YP_009325923 |
| Sugarcane streak Egypt virus | AF037752 | NP_045943 | NP_045942 |
| Sugarcane streak Reunion virus | AF072672 | NP_840051 | NP_840050 |
| Sugarcane streak virus | M82918 | NP_620491 | NP_620490 |
| Sugarcane striate virus | KX352043 | YP_009389275 | YP_009389274 |
| Sugarcane white streak virus | KJ210622 | YP_009026387 | YP_009026386 |
| Sweet potato symptomless virus 1 | KY565237 | YP_009362981 | YP_009362980 |
| Switchgrass mosaic-associated virus 1 | KF806701 | YP_009111308 | YP_009111307 |
| Tobacco yellow dwarf virus | M81103 | NP_620725 | NP_620724 |
| Urochloa streak virus | EU445699 | YP_001941154 | YP_001941153 |
| Wheat dwarf India virus | JQ361910 | YP_006273069 | YP_006273068 |
| Wheat dwarf virus | AJ311031 | NP_542348 | NP_542347 |
