# Supplementary material for: To be or not to be a virus: A novel chimeric circular Rep-encoding single stranded DNA virus with interfamilial gene exchange illustrates the considerable evolutionary capacity of ssDNA viruses
Source: PLoS One. 2025 Aug 18;20(8):e0309278. doi: 10.1371/journal.pone.0309278 (PMC12360566; doi:10.1371/journal.pone.0309278)
Supplement: S3 Table — (DOCX) [file pone.0309278.s005.docx]

**Supplementary Table 3. Primers used for virus indexing**

| **Primer name** | **Primer sequence** | **Amplicon size** | **Hybridization temperature (°C)** | **Targeted virus** |
| --- | --- | --- | --- | --- |
| NV3_F185 | 5’-TGGAAAGTGGTAATTCGCCC-3’ | 876 | 60 | CPMSV |
| NV3_R1061 | 5’-CGACTCCACTCTGAACTTCC-3’ |  |  |  |
| NV4_F241 | 5’-AGCTTTGTCCCTCTACTGGT-3’ | 449 | 60 | CPAV |
| NV4_R690 | 5’AGGTTGTCTTACCCTCTGCT-3’ |  |  |  |
| MSV_F1875 | 5’-GGGACTGACCTGGAAGATGT-3’ | 750 | 60 | MSV |
| MSV_R2625 | 5’-CTATAAAACAAGGAACGGCGG-3’ |  |  |  |
