# Supplementary material for: To be or not to be a virus: A novel chimeric circular Rep-encoding single stranded DNA virus with interfamilial gene exchange illustrates the considerable evolutionary capacity of ssDNA viruses
Source: PLoS One. 2025 Aug 18;20(8):e0309278. doi: 10.1371/journal.pone.0309278 (PMC12360566; doi:10.1371/journal.pone.0309278)
Supplement: S4 Table — (DOCX) [file pone.0309278.s006.docx]

**Supplementary Table 4. Long read sequencing statistics.**

| **Raw reads** | **Passed reads** | **Sample Id** | **Trimmed reads** | **CPMSV reads** | **CPAV reads** | **Length CPMSV assembly** | **Length CPAV assembly** |
| --- | --- | --- | --- | --- | --- | --- | --- |
| 176,330 | 125,778 | 21_REU_E0816_3 | 10,616 | 325 | 1,543 | 2,811 | 2,741 |
|  |  | 21_REU_E0816_4 | 19,498 | 455 | 677 | 2,804 | 2,725 |
|  |  | 21_REU_E0816_5 | 10,352 | 90 | - | 2,797 | - |
